# Risk Factors Related to Operative Duration and Their Relationship With Clinical Outcomes in Pediatric Patients Undergoing Roux-en-Y Hepaticojejunostomy

**DOI:** 10.3389/fped.2020.590420

**Published:** 2020-12-08

**Authors:** Yongjun Zhou, Yunfei Zhang, Hongjie Guo, Chao Zheng, Chunbao Guo

**Affiliations:** ^1^Department of Pediatric General Surgery, Children's Hospital, Chongqing Medical University, Chongqing, China; ^2^Ministry of Education Key Laboratory of Child Development and Disorders, Children's Hospital of Chongqing Medical University, Chongqing, China; ^3^National Clinical Research Center for Child Health and Disorders, Children's Hospital of Chongqing Medical University, Chongqing, China; ^4^China International Science and Technology Cooperation Base of Child Development and Critical Disorders, Children's Hospital of Chongqing Medical University, Chongqing, China; ^5^Chongqing Key Laboratory of Pediatrics, Children's Hospital of Chongqing Medical University, Chongqing, China; ^6^Chongqing Engineering Research Center of Stem Cell Therapy, Children's Hospital of Chongqing Medical University, Chongqing, China; ^7^Department of Anaesthesia, Children's Hospital, Chongqing Medical University, Chongqing, China; ^8^Department of Orthopedics, Children's Hospital of Chongqing Medical University, Chongqing, China

**Keywords:** operative time, Roux-en-Y hepaticojejunostomy, postoperative recovery, perioperative complications, risk factors

## Abstract

**Background:** Operative duration might be important for perioperative morbidity, and its involvement has not been fully characterized in pediatric patients. We identified perioperative variables associated with operative duration and determined their influence on clinical outcomes in pediatric patients.

**Methods:** We retrospectively reviewed 701 patients who underwent elective removal of choledochal cysts followed by Roux-en-Y hepaticojejunostomy. The patients were separated into the long operative time group (>165 min) and short operative time group (<165 min) based on the median operative time (165 min). Propensity score matching was performed to adjust for any potential selection bias. The independent risk factors for operative time were determined using multivariable logistic regression analyses.

**Results:** The operative time was often increased by excision difficulty caused by a larger choledochal cyst size (OR = 1.56; 95% CI, 1.09–2.23; *p* < 0.001), a greater BMI (OR = 1.02; 95% CI, 1.00–1.15; *p* = 0.018), and older age (OR = 1.17; 95% CI, 1.02–1.39; *p* = 0.012) in the multivariate analysis. A long surgical duration was associated with delayed gastrointestinal functional recovery, as measured using the time to first defecation (*p* = 0.027) and first bowel movement (*p* = 0.019). Significantly lower levels of serum albumin were found in the long operative time group than in the short operative time group (*p* = 0.0035). The total length of postoperative hospital stay was longer in patients in the long operative time group (7.51 ± 2.03 days) than in those in the short operative time group (6.72 ± 1.54 days, *p* = 0.006).

**Conclusions:** Our data demonstrated that a short operative time was associated with favorable postoperative results. The influencing factors of operative time should be ameliorated to achieve better outcomes.

## Introduction

In the general surgery literature, it has been shown that particularly for gastroenterological surgery, a longer operative time might result in more postoperative complications (1, 2), such as delayed gastrointestinal function recovery, postoperative intestinal obstruction, and prolonged hospitalization (3, 4). It is therefore recommended that the speed of the surgery be optimized to improve postoperative outcomes, especially for major classical operations. Furthermore, operative time has been used as a measure of competence of surgeons. Specific surgical properties, including operative time, have been well documented in the literature and have been used to determine operative learning curves (5, 6). On the other hand, operations performed in a rush might be associated with increased occurrence of errors during surgery (7, 8).

There are many factors that may potentially impact operative duration, including the actual pathology encountered and the surgeon's operative experience (9). A clear evaluation of the factors associated with operative duration is currently lacking. To assess the impact of operative time on postoperative outcomes, the risk factors that contribute to a longer operative duration must be analyzed. Furthermore, although operative time has been previously evaluated in adult patients, it remains poorly determined for practice among pediatric cohorts.

In the current study, we aimed to identify the risk factors for a longer operative time in a pediatric population. We further compared postoperative recovery and outcomes between patients who had a longer or shorter operative duration.

## Materials and Methods

### Patient Selection

**Figure 1:**
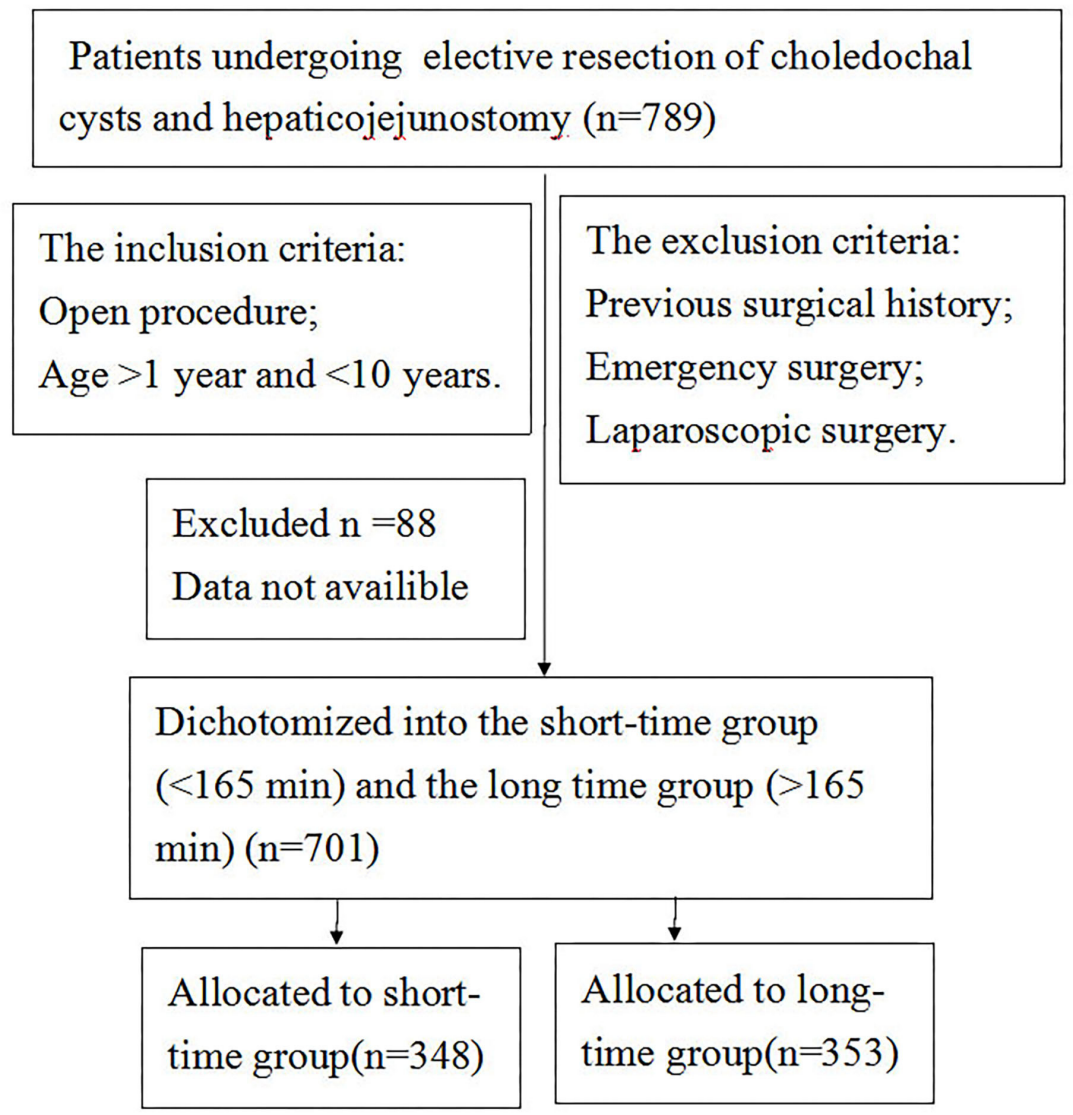
Diagram of the study.

We performed a retrospective analysis of 810 patients who underwent elective choledochal cyst removal followed by Roux-en-Y hepaticojejunostomy at the Department of General Surgery at the Affiliated Chongqing Children's Hospital at Chongqing Medical University between August 2010 and August 2019. The surgical procedures were performed by five qualified and professional attending surgeons. The inclusion criteria were as follows: patients undergoing resection of choledochal cysts and hepaticojejunostomy, open procedure, and age >1 and <10 years. The exclusion criteria were as follows: previous surgical history, coagulopathy or platelet dysfunction, and laparoscopic surgery. The Institutional Review Board of Chongqing Medical University gave expedited approval of this protocol. A flow chart describing the inclusion and exclusion criteria including the ultimate cohort is presented in Figure 1.

### Data Acquisition and Definition

The following data were extracted from the electronic medical records: (1) preoperative variables, including demographic characteristics, clinical parameters, and preexisting comorbidities; (2) intraoperative data, including the American Society of Anesthesiology (ASA) classification, estimated blood loss (EBL), intraoperative hemoglobin levels, intraoperative blood transfusion, operation start time, and duration of the operation (time from skin incision to complete skin closure); and (3) postoperative outcomes, including features of prompt gastrointestinal recovery, postoperative laboratory profiles, and postoperative complications. Gastrointestinal features, including nausea or vomiting, time to normal diet, abdominal bloating and/or cramps, first postoperative flatus or defecation, and gastric retention, were recorded for the first 5 postoperative days. The postoperative complications were ranked according to the Clavien–Dindo classification system. Only grade II complications or higher, including postoperative hemorrhage, wound infection, and intra-abdominal abscesses, were recorded in this research. According to the median operative time (cutoff value, 165 min), patients were dichotomized into the short operative time group (<165 min) and the long operative time group (>165 min) (Table 1) for the purpose of analysis.

**Table 1:** The factors associated with operating time.

	**Total population**		
	**<165 min (348)**	**>165 min (353)**	***p* values**
Age (years)	2.3 ± 1.3	2.9 ± 1.9	0.058
Female: male	93 (26.7)	89 (25.2)	0.41
Weight (kg)	11.9 ± 4.9	12.9 ± 5.3	0.035
BMI, median (range)	24 (20–29)	26 (22–32)	0.023
**Laboratory findings**			
Hypertransaminasemia, *n* (%)	225 (64.7)	236 (66.9)	0.30
Hyperbilirubinemia, *n* (%)	138 (39.7)	143 (40.5)	0.44
WBC (109/L), mean ± SD	9.2 ± 3.5	9.1 ± 3.9	0.17
CRP (mg/L, normal value: 0–8), mean ± SD	12.7 ± 4.3	12.8 ± 4.7	0.29
**Ultrasound presentation**			
Mean CBD, cm, mean ± SD	2.0 ± 0.8	2.6 ± 1.7	0.0012
Charcot's triad, *n* (%)	56 (16.1)	89 (25.2)	0.02
**Operating start time**, ***n*** **(%)**			
Morning	95 (27.3)	126 (35.7)	0.010
Afternoon	253 (72.7)	227 (64.3)	
**ASA classification**, ***n*** **(%)**			
ASA1–2	237 (68.1)	252 (71.4)	0.19
ASA3–4	111 (31.9)	101 (28.6)	

*BMI, body mass index; WBC, white blood cells; CRP, C-reactive protein; CBD, common bile duct; ASA, American Society of Anesthesiology*.

### Propensity Model

Propensity score matching was performed to minimize the bias in baseline characteristics with a multivariable logistic regression model using SPSS 20.0 (IBM, Armonk, NY, US) or R 3.1.2 (The R Foundation for Statistical Computing). A caliper distance of 0.2 was specified for 1:1 propensity score matching using nearest-neighbor analysis. The linear assumption in the PS model was checked using the generalized additive model, thus matching 108 HS patients to 108 controls. The characteristics of the long and short operative time groups were compared after propensity score matching.

### Statistical Analysis

The categorical variables, presented as frequencies with percentages, were compared using the chi-squared test or Fisher's exact test as appropriate. Normally distributed continuous data are expressed as the means ± (standard deviations) and were tested using Student's t test or the Mann–Whitney U test. Nonnormally distributed data are expressed as medians (interquartile ranges) and were analyzed using the Wilcoxon rank-sum test. Multivariate analysis was performed to determine predictors of operative duration by entering the variables with a significance level of *p* < 0.30 in the univariate analysis. The statistical analyses were performed using SPSS 20.0 (IBM, Armonk, NY, USA).

## Results

A total of 789 patients who underwent elective Roux-en-Y hepaticojejunostomy between 2009 and 2019 met the inclusion criteria. A total of 701 patients were included in the final analysis as 88 patients were excluded because their notes could not be obtained (Table 1).

### Factors Associated With Operative Time

In the current study, the average operative time among all 701 patients was 165 min, with a coefficient of variation (COV) of 46.9%. Table 1 summarizes the demographic, clinical, and operation features based on short and long operative durations. As indicated, there were no significant differences regarding some preoperative parameters such as the gender distribution, ASA classification, and prevalence of the hypertransaminasemia and hyperbilirubinemia (*p* > 0.05) (Table 1). Furthermore, some of the preoperative laboratory tests [white blood cells (WBC), C-reactive protein (CRP)] were similar to those of the short operative time group (*p* > 0.05). However, greater degrees of fat (greater weight and BMI) were associated with the operative duration (*p* < 0.01). Interestingly, operations performed in the morning took longer than those performed in the afternoon. A larger choledochal cyst size and worse symptoms (Charcot's triad) often resulted in a longer operative time (*p* < 0.05). Moreover, when analyzing individual surgeons, we discovered that the operative duration was associated with the surgeon's individual performance (*p* < 0.01), with a longer duration for attending surgeon number 5.

### Multivariate Analysis

Three risk factors for the development of a longer operative time were identified (**Table 4**) in the multivariate logistic regression analysis. As shown in **Table 4**, a larger choledochal cyst size was associated with a longer operative time (OR = 1.56; 95% CI, 1.09–2.23; *p* < 0.001). Similar results were discovered for the patients with a greater BMI (OR = 1.02; 95% CI, 1.00–1.15; *p* = 0.018). The patient's age (OR = 1.17; 95% CI, 1.02–1.39; *p* = 0.012) was also significantly associated with a longer operative time (Table 2).

**Table 2:** Multivariate analysis of related factors for longer operating time.

	**OR**	**95% CI**	***p***
Age (years)	1.17	(1.02–1.39)	0.012
Choledochal cyst size	1.56	(1.09–2.23)	<0.001
BMI	1.02	(1.00–1.15)	0.018

### Postoperative Outcomes

We performed PS matching to eliminate systematic differences in baseline characteristics between the patients in the short and long operative time groups. Under PS matching, 268 patients in the short operative time group were matched to 268 patients in the long operative time group. After PS matching, the values of the standardized mean differences for the included continuous and categorical variables were lower and comparable between the two groups (Table 3).

**Table 3:** The inclusion variables in the PS-matching analysis.

	**Total population**		
	**<165 min (268)**	**>165 min (268)**	***p* values**
Age (years)	2.42 ± 1.26	2.51 ± 1.39	0.26
Female: male	87 (37.5)	85 (39.9)	0.41
Weight (kg)	12.1 ± 4.4	12.2 ± 4.8	0.19
BMI, median (range)	25 (22–27)	25 (22–28)	0.32
**Laboratory findings**			
Hypertransaminasemia, *n* (%)	175 (65.3)	176 (65.7)	0.38
Hyperbilirubinemia, *n* (%)	108 (40.3)	111 (41.4)	0.46
WBC (109/L), mean ± SD	9.1 ± 3.4	9.1 ± 3.5	0.32
CRP (mg/L, normal value: 0–8), mean ± SD	12.7 ± 3.7	12.7 ± 3.9	0.37
**Ultrasound presentation**			
Mean CBD, cm, mean ± SD	2.1 ± 0.7	2.3 ± 1.1	0.24
Charcot's triad, *n* (%)	43 (16.0)	44 (16.4)	0.49
**Operating start time**, ***n*** **(%)**			
Morning	81 (30.2)	85 (31.7)	0.45
Afternoon	187 (69.8)	183 (68.3)	
**ASA classification**, ***n*** **(%)**			
ASA1–2	186 (69.4)	183 (68.3)	0.37
ASA3–4	82 (30.6)	85 (31.7)	

*BMI, body mass index; WBC, white blood cells; CRP, C-reactive protein; CBD, common bile duct; ASA, American Society of Anesthesiology*.

Overall, there were no differences in terms of the number of hypokalemic episodes, intraoperative hypotensive events, metabolic acidosis (defined by low bicarbonate), or other laboratory and hemodynamic parameters between the short operative time and long operative time groups (Table 4). A trend for accelerated gastrointestinal function recovery was noted in patients in the short operative time group compared with those in the long operative time group, as indicated by the first defecation (*p* = 0.027) and first bowel movement (*p* = 0.019). Furthermore, the ALB values on postoperative days (PODs) 2–5 were lower in patients who underwent longer procedures (*p* = 0.0035). Patients with longer operative times suffered almost equally from vomiting and diarrhea events as patients with shorter operative times on POD 5 (Table 4). The incidence of abdominal distention within 5 PODs was marginally higher in the patients with longer operative times than in those with shorter operative times (*p* = 0.068).

**Table 4:** Outcome characteristics in the matched population with operative times above and below 165 min.

	** <165 min (268)**	**>165 min (268)**	***p* values**	**Odds ratio (95% CI)**
Hypotensive events, *n* (%)	26 (9.7)	31 (11.6)	0.29	
Norepinephrine usage, *n* (%)	38 (14.2)	41 (15.3)	0.40	
Furosemidum, *n* (%)	24 (9.0)	27 (10.1)	0.38	
Metabolic acidosis, *n* (%)	8 (3.0)	11 (4.1)	0.32	
Nadir of hemoglobin(g/L)	27 (10.1)	38 (14.2)	0.093	
Operative blood loss (ml)				
Intraoperative transfusion, *n* (%)	31 (11.6)	44 (16.4)	0.067	
Albumin on POD 2–5, mean ± SD (g/L, normal range, 35–50)	31.3 ± 5.9	28.6 ± 6.8	0.008	1.12 (1.01–1.56)
First bowel movement, days, Mean ± SD	2.3 ± 0.9	2.8 ± 0.8	0.019	0.56 (0.38–0.98)
Abdominal distension, *N* (%)	26 (9.7)	38 (14.2)	0.068	0.65 (0.38–1.11)
Diarrhea, *N* (%)	13 (4.9)	21 (7.8)	0.11	
Vomiting, *N* (%)	24 (9.0)	32 (11.9)	0.16	
Removal nasogastric tube (days)	3.2 ± 0.4	3.4 ± 0.5	0.51	
First defecation (days)	2.5 ± 1.4	3.4 ± 1.5	0.027	0.69 (0.31–1.07)
No. of patients with complications, *n* (%)	62 (23.1)	83 (31.0)	0.026	0.67 (0.46–0.99)
Total number of complications, *n* (%)	73 (27.2)	94 (35.1)	0.031	0.69 (0.48–1.00)
Postoperative stay (days)	6.7 ± 1.5	7.5 ± 2.0	0.006	0.72 (0.42–0.96)

*POD, postoperative day*.

Finally, there were more postoperative complications in patients in the long operative time group than in those in the short operative time group, including infectious complications, anastomotic leakage, incisional dehiscence, and sepsis. Sixty-two patients (62/268, 23.1%) in the short operative time group experienced at least one complication compared with 83 (83/268, 31.0%) in the long operative time group, with an OD of 0.67 (95% CI, 0.46–0.99; *p* = 0.026) (Table 4). The mean length of postoperative stay was 7.51 ± 2.03 days in patients in the long operative time group, which was significantly longer than that (6.72 ± 1.54 days) in the short operative time group (*p* = 0.006).

## Discussion

We conducted the present analysis in a pediatric surgical cohort following the same surgical procedure to explore optimized operative duration during abdominal surgery. Current data from a tertiary care hospital suggested that several factors were associated with the operative time, including patient age, comorbidity, and mean CBD. Furthermore, a shorter operative duration was associated with significant improvements in recovery measures and postoperative complications. Only comorbid diseases contributed to both longer operative time and surgical outcomes.

The factors that led to a long duration of abdominal operations were often complicated and associated with the case type, surgeon's proficiency and characteristics, emergency operations, and other factors (10, 11). The operative duration may be prolonged by unexpected intraoperative findings and operative complexity. Understanding the risk might delay progression in favor of reducing the operative duration. The current surgical outcomes, including complication rates, were comparable to those calculated in previous reports (12, 13). The pediatric cohort was treated following identical surgical procedures to those originating from university medical centers and was therefore deemed satisfactory for the assessment of the factors influencing operative duration. In this study, we measured the amount of variability in operative duration and identified several factors related to a longer operative duration. After multivariate analysis, the results showed that a larger choledochal cyst size and severe comorbidities (such as Charcot's triad) independently resulted in longer operative times. Another factor was patient age, which had not been shown in previous studies to be associated with a longer operative time during Roux-en-Y hepaticojejunostomy. The reasons for this association are unclear, but they may pertain to alterations in the deeper operative surface related to operative visual field exposure that are beyond the scope of these data set. Furthermore, older patients require thicker anastomoses that need longer operative times to construct, likely reflecting the increased complexity of longer cases. We only enrolled patients undergoing a select operation here and found a relationship between severe comorbidities and operative time, which is likely related to chronic inflammation and the resulting impact on the pericyst tissue. Due to the select nature of this operation, the tissue adhesions were not so severe as to influence the operative time.

Several studies have shown that the experience and distinctive characteristics of the individual surgeon or the surgical team influences the operative duration (14–16). Senior surgeons with increased experience are likely to decrease the duration of procedures. Attention should be paid to a variety of individual surgeon characteristics, such as experience, techniques, personality, age, gender, and surgical preferences. Furthermore, there were many trainees involved in all the operations. The involvement of this information could not be collected from the medical records; thus, research is needed in this area. Improvements in operating process efficiency, including an operative team and an anesthesia team, can reduce operative duration (17, 18). Interestingly, we demonstrated similar findings that operations performed in the morning usually took more time than those performed in the afternoon, which could be the main reason for this finding. The potential impact of surgeon fatigue and human circadian variations reduced the workload requirements based on operative experience; however, an effect has not been demonstrated to date.

A longer operative time is a surrogate marker of surgical difficulty, which might be associated with perioperative morbidity and delayed discharge. Although goal-directed fluid therapy was adopted to control fluid administration, a prolonged operative duration may account for excessive intravenous fluid administration and blood loss (19, 20). In this study, an increased total volume of intraoperative crystalloid administration and blood loss occurred following surgeries with an increased operative duration. Blood transfusion administration was an independent predictor of increased morbidity for surgical patients, including gastroenterology patients (21). Furthermore, fluid accumulation might decrease tissue oxygenation, which is also unfavorable for postoperative gastroenterological recovery and wound healing and the most important reason for the postoperative length of stay (22). Nevertheless, uneventful recovery was the outcome for most choledochal cyst resection patients. This study identified remarkable beneficial effects of a shorter operative duration for postoperative gastrointestinal recovery in terms of defecation and resumption of an oral solid diet. The difficulty of evaluating patients is that clinical intestinal complaints cannot be adequately monitored. Our analysis involved closely monitoring intestinal function in a hospital setting, with frequent nursing assessments and continuous intestinal function monitoring. Therefore, any clinically significant intestinal complaints would likely be captured. In our study, we indeed detected an increased total number of complications and unfavorable postoperative outcomes following increased operative durations, which was consistent with the findings of previous studies (23). A longer operative time may be related to prolonged general anesthesia and the potential for prolonged catheterization, which should explain some unfavorable postoperative outcomes, including increased urinary tract infection (UTI) risk with urinary retention and respiratory infections. A long operative duration might accelerate metabolism and increase the consumption of nutrients, as suggested by previous research (24). In the current study, postoperative ALB values, which have been widely adopted to evaluate the preoperative immunological and nutritional statuses of patients undergoing gastrointestinal and cardiac surgery, were significantly greater in the long operative time group (25, 26). In the present study, for patients with longer operative times, the average length of stay was longer than the average length of stay of those with shorter operative times in our institution, between 4 and 5 days. The present data highlighted the benefits of a short operative duration, which might be associated with postoperative complications in pediatric patients undergoing major surgery. However, while our study supports the notion that several factors were associated with a prolonged operative time, this was not necessarily associated with postoperative complications. Evidence-based best practices should be implemented to promote safer, higher quality patient care.

There are several potential limitations; therefore, in the interpretation of the current data, care should be taken. The retrospective nature of the design with a relatively small size of heterogeneous patients may limit the generalizability of our conclusions. Moreover, selection bias could not be fully eliminated, but PS matching was performed. We were also unable to identify complications or returns to the OR occurring >30 days after the index operations. There have been many practices that have been updated over a long time period of management for many patients, which may not reflect the outcomes from current treatment algorithms. In addition, it is unlikely that there was strict separation of the individual contributions of surgeons, preventing us from determining the differences between individual surgeons. It is important for future studies to investigate the effect of each surgeon individually. Finally, it might be interesting to evaluate the effect of surgical nurses because they are also part of the surgical team and may affect the operative duration.

## Conclusions

In the current study, we characterized risk factors, such as patient comorbidities, surgeon peculiarities, and cyst size, that may result in longer operative times. A long operative duration negatively impacts the postoperative outcomes of pediatric patients undergoing major abdominal operations. Further research is needed to delineate clinical scenarios to optimize preoperative planning and maximize surgical efficiency.

## Data Availability Statement

The original contributions presented in the study are included in the article/supplementary material, further inquiries can be directed to the corresponding author/s.

## Ethics Statement

The studies involving human participants were reviewed and approved by ethics committee of Chongqing Medical University. Written informed consent to participate in this study was provided by the participants' legal guardian/next of kin. Written informed consent was obtained from the individual(s), and minor(s)' legal guardian/next of kin, for the publication of any potentially identifiable images or data included in this article.

## Author Contributions

YZho and YZha designed and analyzed the data. CZ evaluated the manuscript. HG performed the statistical measurements. CG analyzed the data and wrote the paper. All authors contributed to the article and approved the submitted version.

## Conflict of Interest

The authors declare that the research was conducted in the absence of any commercial or financial relationships that could be construed as a potential conflict of interest.

## Abbreviations

ALB: albumin
ASA: American Society of Anesthesiology
BMI: body mass index
CBD: common bile duct
CI: confidence interval
COV: coefficient of variation
CVP: central venous pressure
EBL: estimated blood loss
OR: odds ratio
POD: postoperative day
RBC: red blood cell
UTI: urinary tract infection.
